# Organ of Corti macrophages: a distinct group of cochlear macrophages with potential roles in supporting cell degeneration and survival

**DOI:** 10.3389/fimmu.2025.1617146

**Published:** 2025-06-25

**Authors:** Mengxiao Ye, Celia Zhang, Dalian Ding, Guang-Di Chen, Henry J. Adler, Rania Sharaf, Bo Hua Hu

**Affiliations:** ^1^ Department of Communicative Disorders and Sciences, University at Buffalo, Buffalo, NY, United States; ^2^ Department of Audiology, School of Health Sciences, University of the Pacific, San Francisco, CA, United States; ^3^ Department of Speech, Language and Hearing, University of Texas at Dallas, Dallas, TX, United States

**Keywords:** macrophage, cochlea, organ of Corti, supporting cells, inflammation, galectin-3, ototoxicity, aging

## Abstract

Macrophages are the primary immune cells in the cochlea, essential for maintaining cochlear homeostasis and orchestrating inflammatory responses to pathological events. Although these cells have been found in various parts of the cochlea, their presence in the organ of Corti, a critical structure for acoustic sensing, remains poorly understood. The present study was designed to examine macrophage responses to ototoxic drug-induced cochlear damage and age-related cochlear degeneration, with a particular focus on the pathological conditions that trigger macrophage recruitment into the organ of Corti. We used a model of ototoxicity induced by cyclodextrin, a cyclic oligosaccharide known for its ability to induce rapid sensory cell damage at high doses. Cochlear tissues were collected for macrophage assessment. Multiple protein markers, including CD45, Iba1, galectin-3, CD68, myosin 7α, and IFIT3, were used to label sensory cells, macrophages, and supporting cells in cochlear sensory epithelia. Consistent with previous reports, our study confirms that macrophages are absent from the organ of Corti in mouse cochleae under normal conditions and during the acute phase of cochlear damage. However, macrophages enter the organ of Corti during the chronic phase of cochlear pathogenesis. These macrophages exhibit an activated state and display a distinct functional profile compared to macrophages outside the organ of Corti. Importantly, we demonstrate that the organ of Corti macrophage activity is not directly related to the process of sensory cell degradation. Instead, their activity is associated with supporting cell pathogenesis. Moreover, our study shows that the organ of Corti macrophages are also present in aging mouse cochleae. Collectively, our findings reveal the conditions that lead to macrophage recruitment into the organ of Corti and their involvement in supporting cell survival and degeneration. These findings provide valuable insights for future strategies focused on modulating macrophage activity to reduce tissue damage and promote repair in the cochlea following injury.

## Introduction

The cochlea plays a pivotal role in hearing perception. Central to its function is the organ of Corti, a specialized structure housing sensory cells and supporting cells. This hearing organ is highly susceptible to various pathological conditions, such as acoustic trauma, ototoxicity, genetic mutations, and aging degeneration. Pathogenesis of the organ of Corti is a major cause of sensory hearing loss.

The cochlea has a robust immune capacity. It is well established that the cochlea contains resident immune cells under resting conditions, with macrophages being the primary immune cell population within the cochlea (1–4). These macrophages are located in various parts of the cochlear structure, including the lateral wall, luminal surface of the perilymph spaces, neural regions in the modiolus, and osseous spiral lamina (1, 3, 5). They provide essential immune surveillance to maintain cochlear homeostasis and function under normal conditions. Upon pathogenesis, the cochlea recruits circulating immune cells, primarily monocytes, into the cochlea (6–10). These infiltrating monocytes differentiate into macrophages and play key roles in various aspects of the inflammatory response, including the production of inflammatory molecules, phagocytosis of damaged cells, tissue repair, and antigen processing (6–10).

While previous studies have documented the presence of macrophages in the cochlea, reports specifically focusing on macrophages in the organ of Corti remain scarce. This is mainly because most prior studies have shown that the organ of Corti typically lacks immune cells under normal conditions (1, 11, 12), except for during the early postnatal period (13). This absence of macrophages is not surprising, as large, mobile macrophages within the organ of Corti could affect its mechanical properties, potentially impairing its vibrations and, consequently, hearing. However, the absence of immune cells does not imply that the organ of Corti lacks immune capacity. Our previous work demonstrated that supporting cells within the organ of Corti produce immune molecules such as toll-like receptors, which regulate cochlear immune responses to acoustic injury (14). Supporting cells also participate in cochlear responses to viral infections, contributing to the protection of hair cells (15–17). In addition, these cells are capable of phagocytosing damaged sensory cells (4, 18–23), express a wide range of immune molecules, and participate in inflammatory responses following cochlear damage (4, 24–27).

Reports on macrophages in the organ of Corti following cochlear damage remain limited and inconsistent. Ladrech and colleagues identified macrophages in the organ of Corti in rat cochleae following amikacin-induced ototoxicity (28). Similarly, macrophages have been reported in the organ of Corti in rodent cochleae following acoustic injury (1, 29), as well as in human patients with posterior cranial fossa meningiomas (30). However, in our previous observations, macrophages were not detected in the organ of Corti of several cochlear damage models, including acoustic injury and age-related degeneration (8, 10, 31). This discrepancy suggests that macrophage recruitment into the organ of Corti is not a common immune response but may occur only under specific pathological conditions. At present, the mechanisms and pathological conditions that trigger macrophage infiltration into the organ of Corti remain unclear. Another important question is whether macrophages that infiltrate the organ of Corti exhibit distinct phenotypes compared to those in other regions. These differences may reflect specialized functions adapted to their local environment. Given the critical role of macrophages, addressing these questions is essential for understanding how the cochlear immune system responds to cochlear damage.

The present study was designed to examine macrophage responses to ototoxic drug-induced cochlear damage and age-related cochlear degeneration, with a particular focus on macrophages within the organ of Corti. We used a model of ototoxicity induced by cyclodextrin, a cyclic oligosaccharide known for its ability to solubilize lipophilic substances. While cyclodextrin is considered safe at low doses, high doses are ototoxic, causing rapid sensory cell damage in the cochlea (32–35). We selected cyclodextrin to induce ototoxicity due to its mild systemic toxicity (33, 34), making it an appropriate choice for chronic studies of inner ear damage.

## Materials and methods

### Subjects

This study utilized 24 young, healthy CBA/CaJ mice (22–28 grams, 1.5 to 4 months old) and 6 aged C57BL/6J mice (17 to 19 months old), with an equal number of males and females, purchased from the Jackson Laboratory (Bar Harbor, ME, USA). In the current study, we included both male and female mice to ensure that our findings are broadly applicable across sexes. All mice were housed at the University at Buffalo Laboratory Animal Facility, employing a light cycle of 12 hours on and 12 hours off (6 am to 6 pm). Three to four mice of the same sex were housed per cage. The temperature and humidity of the room housing the animals were maintained at 22°C and 40-60% humidity, respectively.

The cyclodextrin ototoxicity study utilized CBA/CaJ mice, which were randomly assigned to experimental groups based on tissue collection time points: 6 mice in the acute damage group (tissues collected at 1–4 days post-treatment) and 10 mice in the chronic damage group (tissues collected from 2 mice at 2 weeks and from 8 mice at 6–10 weeks post-treatment). An additional 8 mice were used as controls. C57BL/6J mice (*n*=6) were used to examine age-related changes and did not receive any cyclodextrin treatment. The number of mice or cochleae used for each assessment parameter will be detailed in the Results section. The Institutional Animal Care and Use Committee of the State University of New York at Buffalo approved all procedures involving the use and care of animals.

### Induction of cyclodextrin ototoxicity

2-Hydroxypropyl-β-cyclodextrin (referred to as cyclodextrin throughout this manuscript; Cayman Chemical Company, Cat # 16169, Ann Arbor, MI, USA) was freshly prepared using normal saline and administered as a single subcutaneous injection at a dose of 8 mg per gram of body weight, with a total volume of 0.3 ml, injected at the midline of the back. The control mice received the same volume of saline solution only. The overall health of the mice was monitored daily during the first week and then twice a week until the time of sacrifice.

### Cochlear tissue collection

CBA/CaJ mice were sacrificed at different time points following cyclodextrin treatment. For the acute damage group, the mice were sacrificed at 1–4 days after the treatment (*n*=6 mice). For the chronic damage group, mice were sacrificed at 2 weeks (*n*=2 mice) or at 6 to 10 weeks after treatment (*n*=8 mice). Lastly, the mice in the control group (*n*=8 mice) were sacrificed at the same time as those in the experimental group. Specifically, 3 mice were sacrificed alongside the acute group (1–4 days post-treatment), and 5 mice were sacrificed alongside the chronic group (6–10 weeks post-treatment). C57BL/6J mice were sacrificed at 17 to 19 months (*n*=6 mice). All the mice were sacrificed using CO_2_ asphyxiation, followed by decapitation. The cochleae were quickly removed from the skull and fixed overnight with 10% buffered formalin at 4°C. The cochleae were dissected in 10 mM phosphate-buffered saline (PBS) for subsequent analyses.

### Assessment of outer hair cell damage

Before preparing whole-mount cochlear sensory epithelia for detailed examination using confocal microscopy, we performed an *in situ* OHC quantification for cochleae in the acute damage group (*n*=5 cochleae, 3–4 days post-treatment) and the chronic damage group (*n*=8 cochleae, 6–8 weeks post-treatment). This *in situ* observation reduced the possibility of dissection-induced damage. Specifically, the apical bony shell of the cochlea was removed using a fine-tip diamond drill to expose the apical section of the cochlea. The cochleae were stained with Alexa Fluor 488-labeled phalloidin (1:100, Cat# A12379, Invitrogen, Waltham, MA, USA) in 10 mM PBS for 30 min at room temperature. The stained tissues were photographed *in situ* using an epifluorescence illumination microscope (Z6 APO apochromatic zoom system) equipped with a digital camera (DFC3000 G microscope camera) controlled by Leica Application Suite V4 PC-based software (Leica Microsystems). After imaging hair cells in the apical region, the apical turn of the cochlea was removed to expose the middle and basal turns. The sensory epithelium was then imaged section by section to capture hair cells in the middle and basal regions. The sections were stitched together using Adobe Photoshop (Adobe Inc., San Jose, CA, USA) to form a panoramic view of the sensory epithelium. The number of missing OHCs was quantified along the cochlear spiral at intervals of 150 µm, and the results were presented as a cochleogram.

### Whole-mount sensory epithelium preparations

After hair cell quantification, the remaining bony shell of the cochlea was removed using a fine-tip diamond drill. The cochlea was then decalcified with 10% ethylenediaminetetraacetic acid (EDTA) at 4°C for 1 day. After rinsing with 10 mM PBS, the sensory epithelia were removed and stored in 10 mM PBS before staining.

### Immunohistochemistry for immune cells and supporting cells

The current study used multiple protein markers to label macrophages and supporting cells in the cochlea (**Table 1**). Immunolabeling of CD45 protein, a pan-leukocyte marker, was used to visualize immune cells. Macrophages were identified based on their expression of ionized calcium-binding adapter molecule 1 (Iba1), a macrophage-specific calcium-binding protein that has been used in previous studies for identifying cochlear macrophages (1, 11). Galectin-3, a marker for macrophage activation and tissue repair (36–38), was used to assess the functional state of macrophages and supporting cells. CD68, a phagocytic marker, was utilized to evaluate the phagocytic activity of macrophages. Myosin 7α was used to identify sensory cells. Interferon-induced protein with tetratricopeptide repeats 3 (IFIT3), which is highly expressed in the central cytoskeletal bundles of Deiters’ cells and pillar cells (39), was used as a marker for assessing the structural integrity of supporting cells.

**Table 1:** Antibodies and molecular probes used to identify target molecules.

Probe	Targets	Catalog number	Company
CD45 Antibody	Pan-leukocytes	AF114	R&D System
Iba1 Antibody	Macrophages	ab178846	Abcam Inc.
CD68 Antibody	Macrophages	MCA1957GA	Bio-Rad Laboratories
Galectin-3 Antibody	Macrophages and Hensen’s cells	AF1197	Novus Biologicals
IFIT3 Antibody	Deiters’ cells and pillar cells	PA522230	Invitrogen
Myosin 7α Antibody	Hair cells	PA1-936	ThermoFisher Scientific
DAPI	Nuclei	62248	ThermoFisher Scientific
Alexa Fluor^TM^ 488 Phalloidin	Actin	A12379	ThermoFisher Scientific
Alexa Fluor^TM^ 594 Donkey anti-Goat IgG	CD45 Antibody, Galectin-3 Antibody	A11058	ThermoFisher Scientific
Alexa Fluor^TM^ 647 Donkey anti-Rabbit IgG	Iba1 Antibody, CD68 Antibody, IFIT3 Antibody Myosin 7α	A31573	ThermoFisher Scientific

After dissection, whole-mount preparations were incubated in a 0.5% Triton X-100 solution containing 10% donkey or goat serum in PBS (pH 7.4) for 30 minutes at room temperature to permeabilize the cells. The tissues were subsequently incubated overnight at 4°C with one or two selected primary antibodies (see the Results section for details). After incubation with primary antibodies, the tissues were rinsed 3 times in PBS and incubated in the dark with one or two secondary antibodies (see **Table 1** for secondary antibodies) for 2 hours at room temperature.

### Confocal microscopy

All confocal images were obtained using an Andor Dragonfly spinning disk confocal microscope (Oxford Instruments, UK) mounted on an inverted Leica microscope base. Image visualization and acquisition were performed using a 40× water immersion lens with a numerical aperture of 1.3. All images were captured using identical parameter settings, including laser intensity and spectrum, exposure time, zoom size, and step size. Each image covered approximately 300 × 300 µm², encompassing the full thickness of the sensory epithelium from the reticular lamina to the basilar membrane.

The sensory epithelium spiral was segmented into three sections for imaging: the apical section (approximately 0–40% of the distance from the apex), the middle section (approximately 41–85% of the distance from the apex), and the basal section (approximately 86–100% of the distance from the apex), with 2 to 4 images collected for each section.

### Image analysis

Collected images were examined and analyzed using Imaris software (version 10.2, Oxford Instruments, UK). The software’s three-dimensional (3D) visualization feature allowed adjustment of the viewing angle for better visualization. The tissues were digitally sectioned to examine structures of interest located deep within the organ of Corti. The section thickness was adjusted to optimize the visibility of structural details.

### Quantitative analysis of macrophages and their distribution

Macrophages were identified based on their expression of CD45 and Iba1, as well as on their distinct morphology. These cells are typically larger than other immune cells and exhibit unique morphologies, including dendritic, amoeboid, curvilinear, or irregular shapes with projections. The number of macrophages was quantified to assess the inflammatory response after cyclodextrin treatment.

To determine macrophage distribution, we examined surface preparations of the sensory epithelia. Positively stained macrophages were classified into two groups based on their location relative to the basement membrane, a thin layer of extracellular macromolecules within the basilar membrane that separates the cells of the organ of Corti from the underlying mesothelial cells. Specifically, macrophages located above the basement membrane within the organ of Corti were designated as organ of Corti macrophages (OC macrophages). In contrast, macrophages situated beneath the basement membrane on the scala tympani side were classified as basilar membrane macrophages (BM macrophages). This distinction was made using cross-sectional views of 3D confocal images generated with Imaris software.

### Quantitative analysis of immunostaining intensity

To evaluate the functional state of macrophages, we measured the mean gray value of galectin-3 and CD68 immunostaining within individual cells. Cell boundaries were manually traced using Adobe Photoshop (Adobe Inc., San Jose, CA, USA), and gray values were obtained using the Record Measurement function. The values from individual macrophages were averaged for each condition and then compared across different conditions (see the Results section for details).

### Data analysis

Statistical analyses were performed using OriginPro (OriginLab, Northampton, MA, USA) or SigmaPlot (version 10.0.1.25, San Jose, CA, USA). Group means were statistically compared with either a one- or two-tailed Student’s *t* test or a one-way or two-way ANOVA (see Results section for details). An α-level of 0.05 was chosen to denote significance for all statistical tests.

Our preliminary analysis did not reveal significant sex differences in cochlear pathogenesis and macrophage responses following cyclodextrin treatment. Therefore, we combined data from both sexes in our subsequent analyses. All data are presented as mean ± 1 standard deviation. Normative data and equal variance tests were performed for all statistical analyses. If these two criteria were not satisfied, non-parametric tests were performed. Multiple comparisons were performed using Šídák’s, Tukey’s or Dunnett’s multiple comparisons test. Sample sizes were determined using G*Power (version 3.1.92) if similar data from previous studies were available. Selected sample sizes gave 80% power to detect biologically significant changes. If no data were available for power analysis, we estimated the sample size based on pilot observations. If estimated sample sizes exceeded our experimental capacity, we used a sample size that allowed us to determine a trend of changes or differences.

## Results

### Cyclodextrin treatment causes rapid induction of sensory cell death

**Figure 1:**
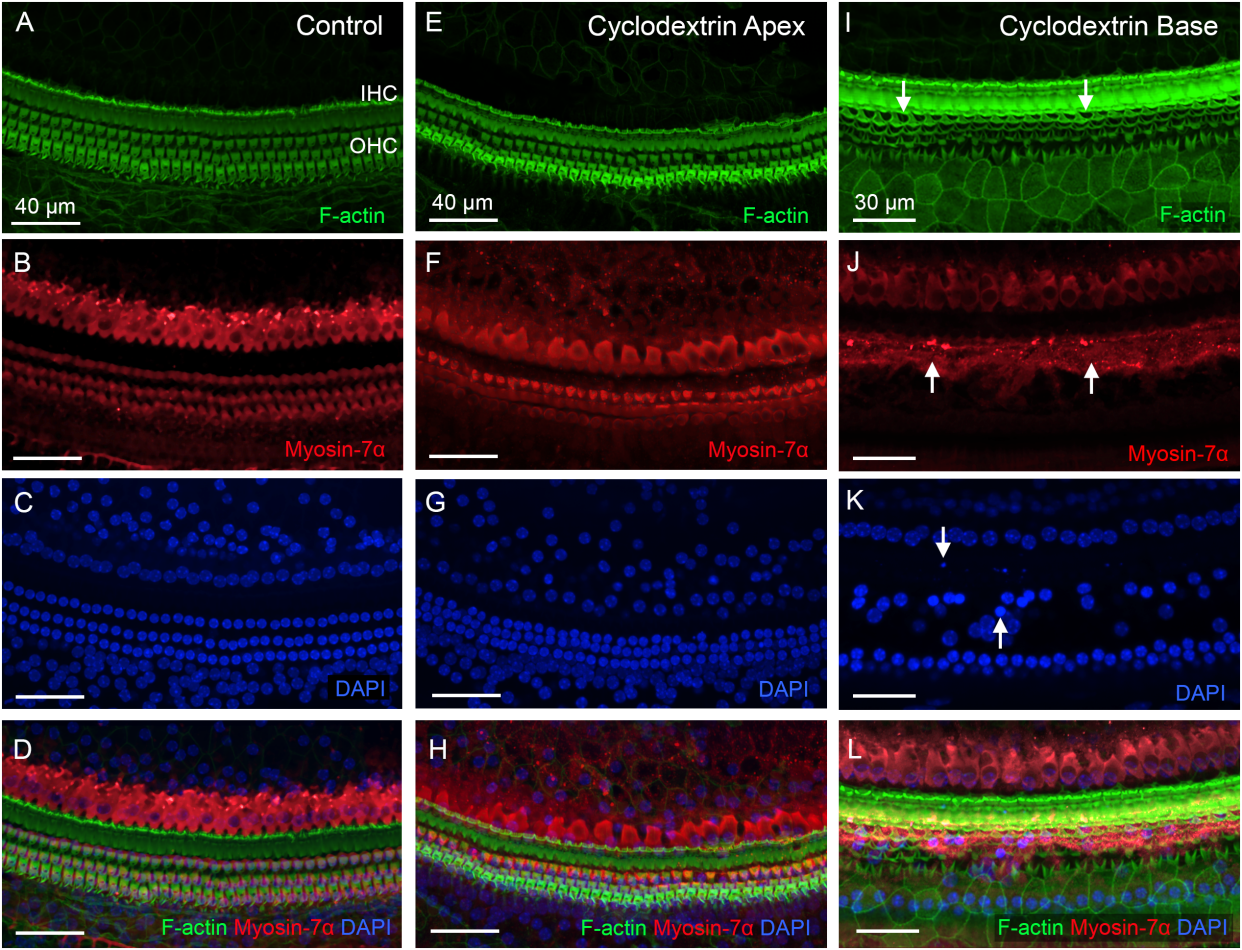
Cyclodextrin treatment induces rapid sensory cell death in mouse cochleae. **(A–D)** Sensory cells in a control cochlea stained for F-actin **(A)**, Myosin-7α **(B)**, and DAPI **(C)**. Both OHCs and IHCs are intact. **(E–H)** Typical image of hair cells in the apical region of the cochlea (25% from the apex) examined at one day post-cyclodextrin treatment. Most hair cells are present. **(I–L)** Significant hair cell death was observed at the basal end of the cochlea at one day post-cyclodextrin treatment. Arrows in **(I)** point to regions of OHC cuticular plates that lack F-actin staining, suggesting cell loss. Arrows in **(J)** indicate fragmented OHC bodies illustrated by Myosin-7α staining. Arrows in **(K)** show condensed or fragmented nuclei in DAPI staining.

We started by examining sensory cell integrity in cyclodextrin-treated cochleae to provide a context for analyzing macrophage responses to cochlear damage. In the control cochleae, hair cells were well organized in the organ of Corti (**Figures 1A-D**). In contrast, significant hair cell death was observed after cyclodextrin treatment. At one day post-treatment, hair cells in the apex of the cochlea appeared largely unaffected (**Figures 1E-H**). However, severe damage was evident in the basal end of the cochlea (**Figures 1I-L**). Phalloidin staining revealed an absence of F-actin on the cuticular plates of OHCs, indicating hair cell loss. Myosin-7α staining displayed fragments of OHC bodies. DAPI staining showed nuclear condensation, fragmentation, and areas of missing OHC nuclei. Inner hair cell (IHC) loss was also observed, but to a lesser extent.

**Figure 2:**
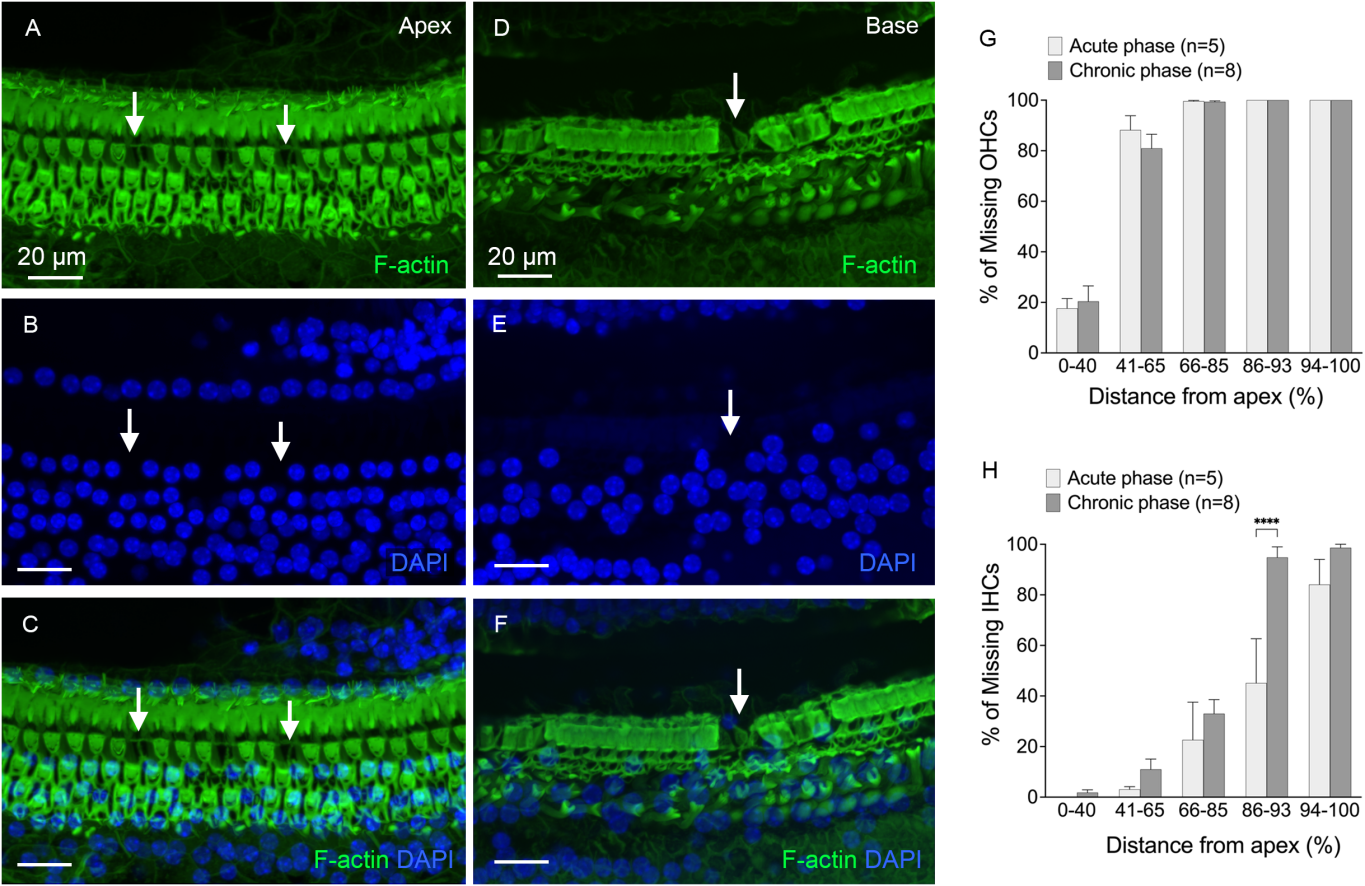
Hair cell loss at the chronic phases of cyclodextrin-induced ototoxicity. **(A-C)** Typical image of the apical region of the cochlea (approximately 25% from the apex), showing areas of hair cell loss (indicated by arrows) in a cochlea examined 6 weeks after cyclodextrin treatment. **(D-F)** Representative image of the basal region of the cochlea (approximately 85% from the apex), taken 6 weeks after cyclodextrin treatment. The image shows complete hair cell loss, with areas of supporting cell loss also evident (indicated by the arrow). **(G, H)** Quantification of missing OHCs **(G)** and IHCs **(H)** at the acute phase (3–4 days) and chronic phase (6–10 weeks) of cyclodextrin treatment. OHC loss spans the middle and basal regions (41–100% from the apex), while IHC loss is confined to the basal region (86–100% from the apex). The severity of OHC loss remains consistent between the acute and chronic phases. However, IHC loss significantly increases in the transition area between the mild and severe IHC loss regions during the chronic phase. **** in indicates *p* < 0.0001.

Hair cell pathologies were also examined during the chronic phase of cyclodextrin-induced ototoxicity (**Figures 2A-F**). **Figures 2G, H** show the distribution and level of OHC and IHC loss at the acute phase (3 to 4 days) and chronic phase (6 to 10 weeks) post-treatment. In the acute phase, OHC loss was primarily observed in the middle and basal regions of the cochlea (41-100% distance from the apex, **Figure 2G**), while IHC loss was primarily confined to the basal end of the cochlea (86-100% distance from the apex, **Figure 2H**). There was only a slight increase in OHC loss in the chronic phase compared to the acute phase of cochlear damage. In contrast, the level of IHC loss markedly increased in the 86-93% distance from the apex (Two-way ANOVA, *F* (4, 55) = 3.679, *p* = 0.01, Šídák’s multiple comparisons test, acute phase *vs*. chronic phase, *p* < 0.0001), indicating continuous progression of IHC loss in the basal portion of the cochlea. In addition to hair cell pathology, we observed supporting cell loss in the basal end of the cochlea (**Figures 2D-F**). A detailed description of this supporting cell loss will be provided in the section below, under “Chronic Pathogenesis of Supporting Cells.” Together, these observations show that a single dose of cyclodextrin treatment causes significant hair cell loss in the cochlea, providing a valuable model for studying macrophage responses.

### Macrophages in the cochlea under normal conditions

**Figure 3:**
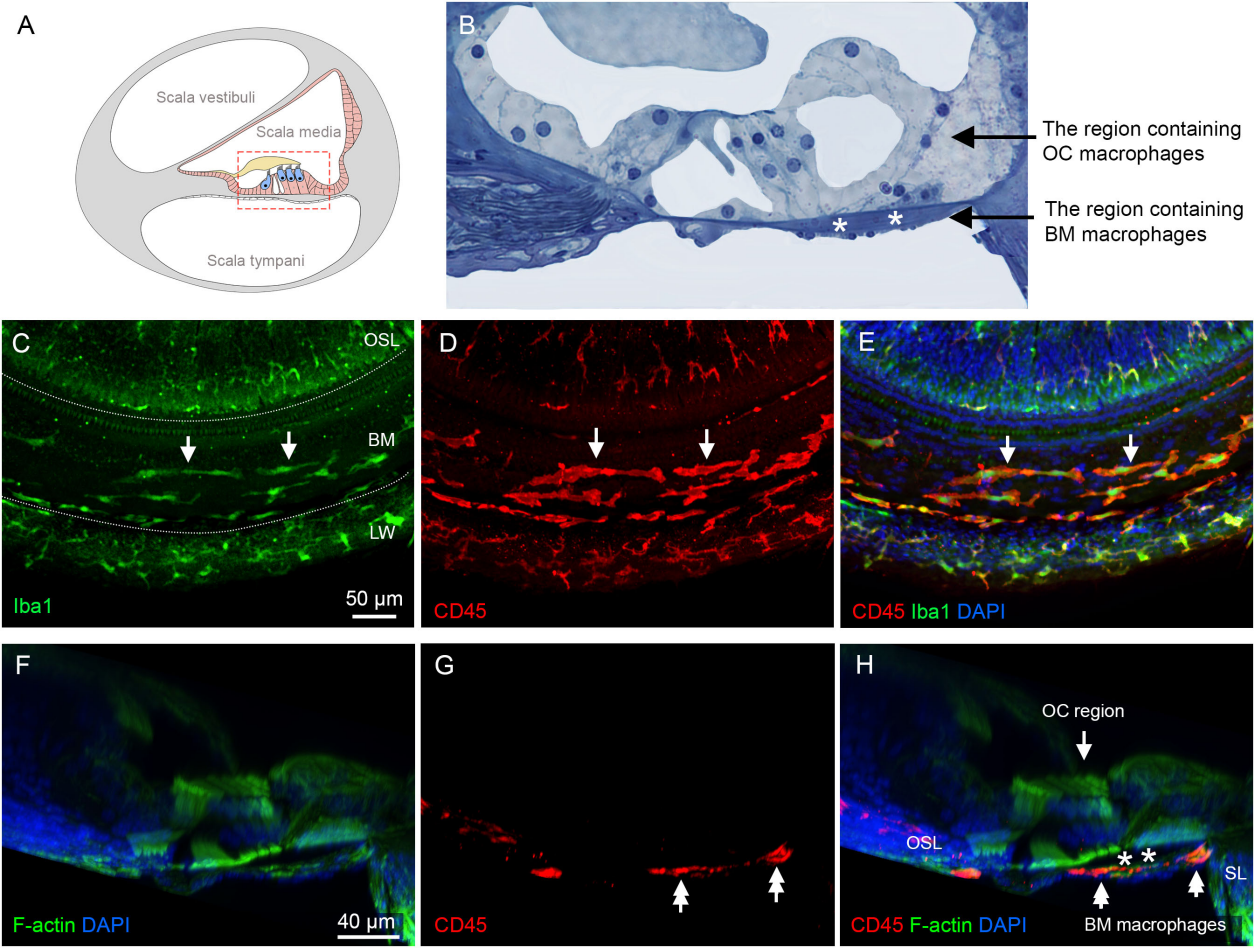
Macrophages in the cochlea under normal conditions. **(A)** A schematic illustration of a cross-sectional view of the cochlea, showing the organ of Corti and its surrounding tissues. The red dotted box highlights the regions of macrophage observation: the organ of Corti and the basilar membrane. **(B)** An enlarged histological view of the areas of macrophage observation. The dark space marked by the white asterisks indicates the basement membrane, which separates the OC macrophages from the BM macrophages. **(C)** Iba1 immunostaining in the middle region of the cochlea from a whole-mount preparation, showing the regions of the osseous spiral lamina (OSL), basilar membrane (BM), and lateral wall (LW). The two dotted lines mark the boundaries of the basilar membrane. Note that Iba1-positive immune cells are present in these regions (arrows). **(D)** Double staining of CD45 in the same tissue as shown in panel **(C, E)** Merged view of **(C, D)** showing that CD45-positive cells also exhibit Iba1 immunoreactivity, indicating that these cells are macrophages. **(F–H)** A cross-sectional view of the whole-mount tissue confirms that the macrophages observed in the basilar membrane (double arrows) are located on the scala tympani side of the basilar membrane. The organ of Corti is devoid of macrophages. The dark space marked by the asterisks indicates the basement membrane, which separates the OC macrophages from the BM macrophages. BM: basilar membrane, OC, organ of Corti; OSL, osseous spiral lamina; SL, spiral ligament; LW, lateral wall.

Our study focused on investigating macrophages within the organ of Corti. These cells are defined as macrophages located in the region between the inner and outer spiral sulcus of the organ of Corti, above the basement membrane (**Figures 3A, B**). These cells are referred to as OC macrophages throughout the study. We also examined macrophages located on the scala tympani side of the basilar membrane, referred to as BM macrophages. These cells served as reference immune cells to demonstrate differences in functional status between OC macrophages and non-OC macrophages. Although BM macrophages are situated near the organ of Corti, they are separated from OC macrophages by the basement membrane. Our subsequent analyses focused on these two distinct macrophage populations.

We first examined normal cochleae. In whole-mount sensory epithelium preparations, Iba1-positive cells were observed in the scala tympani side of the basilar membrane, the lateral wall, and the neural region within the osseous spiral lamina (**Figure 3C**). Further immunostaining of CD45, a pan-leukocyte marker, revealed that all CD45-positive cells displayed Iba1 immunoreactivity (**Figures 3D, E**), consistent with our previous observation that macrophages are the major immune cell population in the cochlea (8, 40).

To differentiate OC macrophages from BM macrophages, we carefully analyzed 3D images collected using confocal microscopy and viewed with Imaris software. As shown in the cross-sectional view of the organ of Corti (**Figures 3F-H**), macrophages are present exclusively on the scala tympani side of the basilar membrane. This observation aligns with our previous findings that the organ of Corti lacks macrophages in normal conditions (10, 41).

### The organ of Corti lacks macrophage activity during the acute phase of cyclodextrin ototoxicity

**Figure 4:**
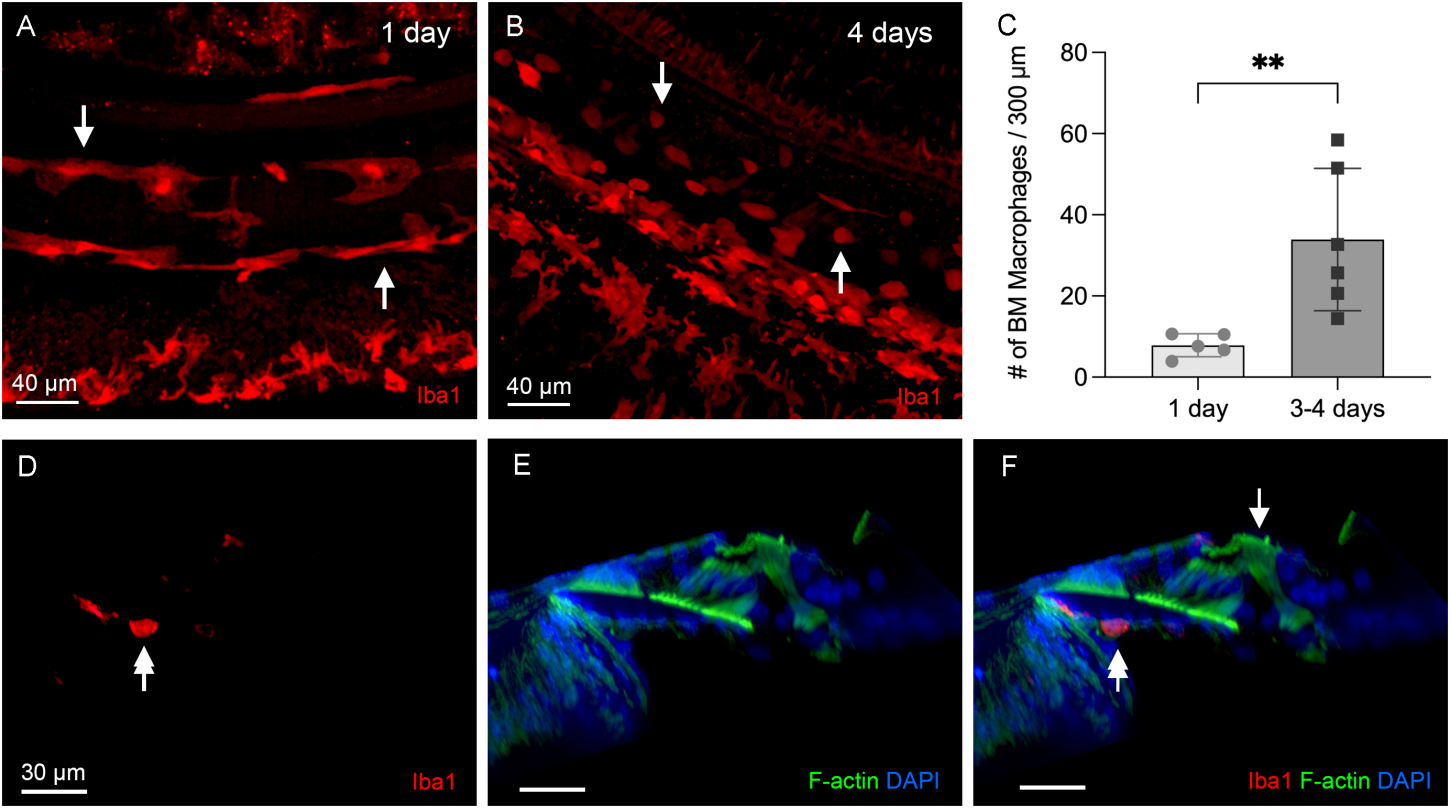
Macrophage activity during the acute phase of cyclodextrin ototoxicity. **(A)** A typical image of BM macrophages stained with Iba1 in the middle section of the cochlea at 1 day post-treatment. At this early stage, BM macrophages retain their normal elongated morphology (arrows). **(B)** A representative image of BM macrophages observed 4 days post-treatment. Numerous small, round Iba1-positive cells are present on the basilar membrane (arrows). These cells are infiltrating monocytes. **(C)** Quantification of Iba1-positive cells reveals a significant increase in BM macrophages during the acute phase of cyclodextrin ototoxicity. ** indicates *p* < 0.01. **(D–F)** A side view of the sensory epithelium shows that Iba1-positive cells (double arrows) are confined to the basilar membrane. Macrophages remain absent in the organ of Corti (arrow) at this acute stage of the damage.

To determine macrophage activity during the acute phase of cyclodextrin ototoxicity, we examined the cochleae at 1, 3, and 4 days after the treatment. At day 1, when significant OHC damage was already evident, resident macrophages on the scala tympani side of the basilar membrane appeared to maintain their resting morphology, characterized by a long and branched morphology (**Figure 4A**). In contrast, at 3–4 days after cyclodextrin treatment, many small, round Iba1-positive cells were identified (**Figure 4B**). Further quantification of BM macrophages in the middle region of the cochlea revealed a significant increase in macrophage number (**Figure 4C**, two-tailed unpaired Student’s *t* test, *t* (9) = 3.257, *p* = 0.0099, *n*=11 images from 4 cochleae), confirming the heightened macrophage activity during the acute phase of cyclodextrin ototoxicity. Noticeably, even with a marked infiltration of macrophages, no macrophages were identified in the organ of Corti (**Figures 4D-F**).

**Figure 5:**
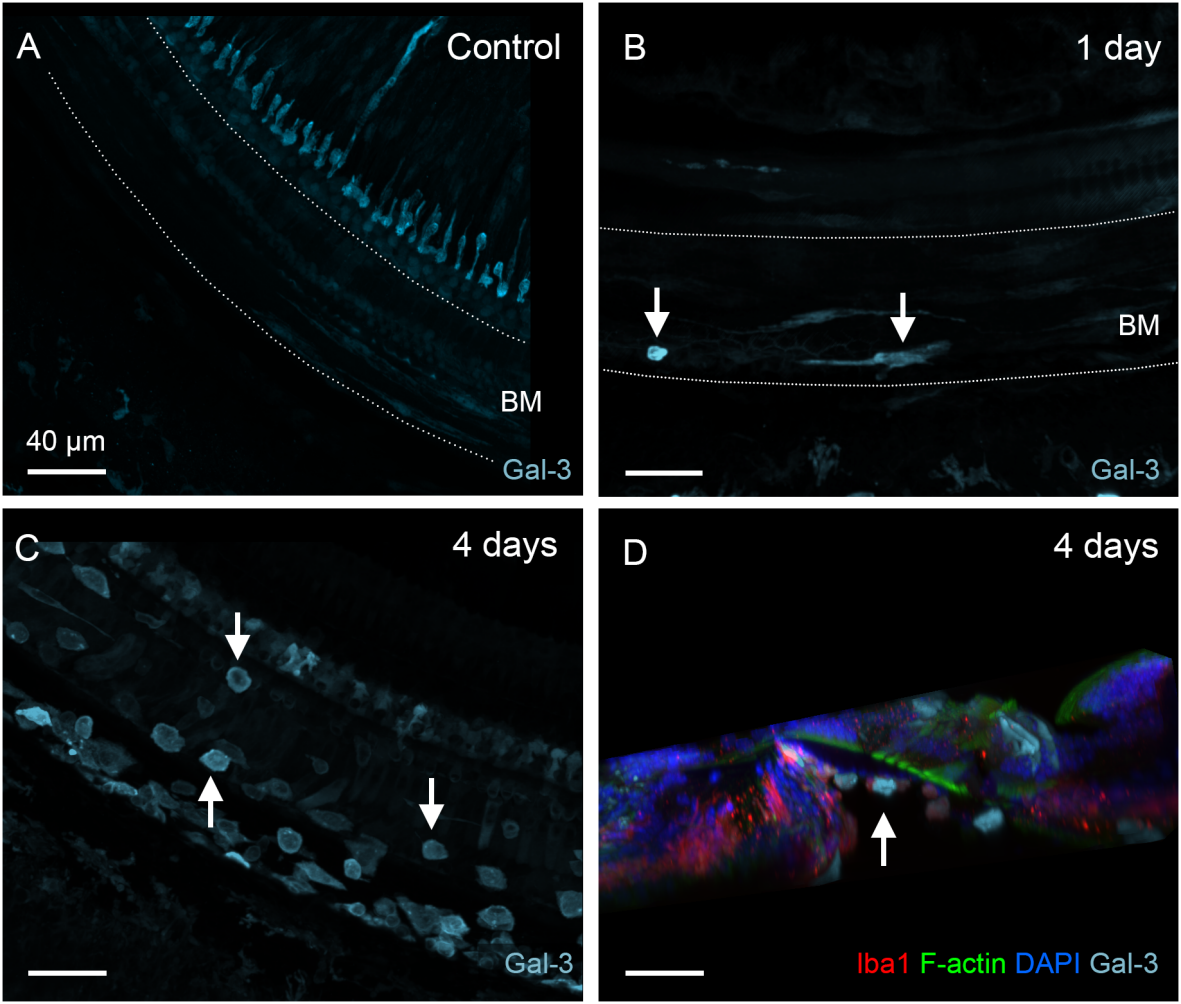
Galectin-3 expression in BM macrophages under normal conditions and during the acute phase of cochlear damage. **(A)** Galectin-3 immunoreactivity in the middle section of a normal cochlea. Macrophages in the basilar membrane region (marked by the dotted line) exhibit little, if any, galectin-3 immunoreactivity. **(B)** A few galectin-3 positive macrophages (arrows) are present in the basilar membrane (marked by the dotted line) 1 day post-treatment. **(C)** A significant increase in the number of galectin-3 positive macrophages (arrows) on the basilar membrane 4 days after cyclodextrin treatment. Notably, many galectin-3-positive cells are small and round, suggesting they are infiltrating monocytes. **(D)** A side view of the sensory epithelium shows that all galectin-3 and Iba1 double-positive cells (arrow) are confined to the basilar membrane. BM: basilar membrane.

To determine the functional state of macrophages during the acute stage of cyclodextrin damage, we examined the expression of galectin-3, a β-galactoside-binding lectin used as a marker for macrophage activation (36, 38, 42, 43). In normal cochleae, BM macrophages exhibited weak or undetectable galectin-3 immunoreactivity (**Figure 5A**). One day after cyclodextrin treatment, galectin-3 expression remained low, with the exception of a few macrophages exhibiting increased galectin-3 immunoreactivity (**Figure 5B**). In contrast, BM macrophages observed at 3–4 days post-cyclodextrin treatment exhibited strong galectin-3 immunoreactivity (**Figure 5C**). Moreover, macrophages in the lateral wall of the middle to basal sections of the cochlea also displayed increased galectin-3 immunoreactivity (image not shown). Notably, none of the galectin-3 positive macrophages were located inside the organ of Corti (**Figure 5D**). Galectin-3 immunoreactivity was also observed in supporting cells, and the changes in its expression among these cells will be discussed below. Together, these observations revealed robust macrophage activity at 3–4 days after cyclodextrin treatment. However, this increased macrophage activity was confined to regions outside the organ of Corti.

### Recovery of BM macrophage activity toward normal levels during the chronic phase of cyclodextrin-induced ototoxicity

**Figure 6:**
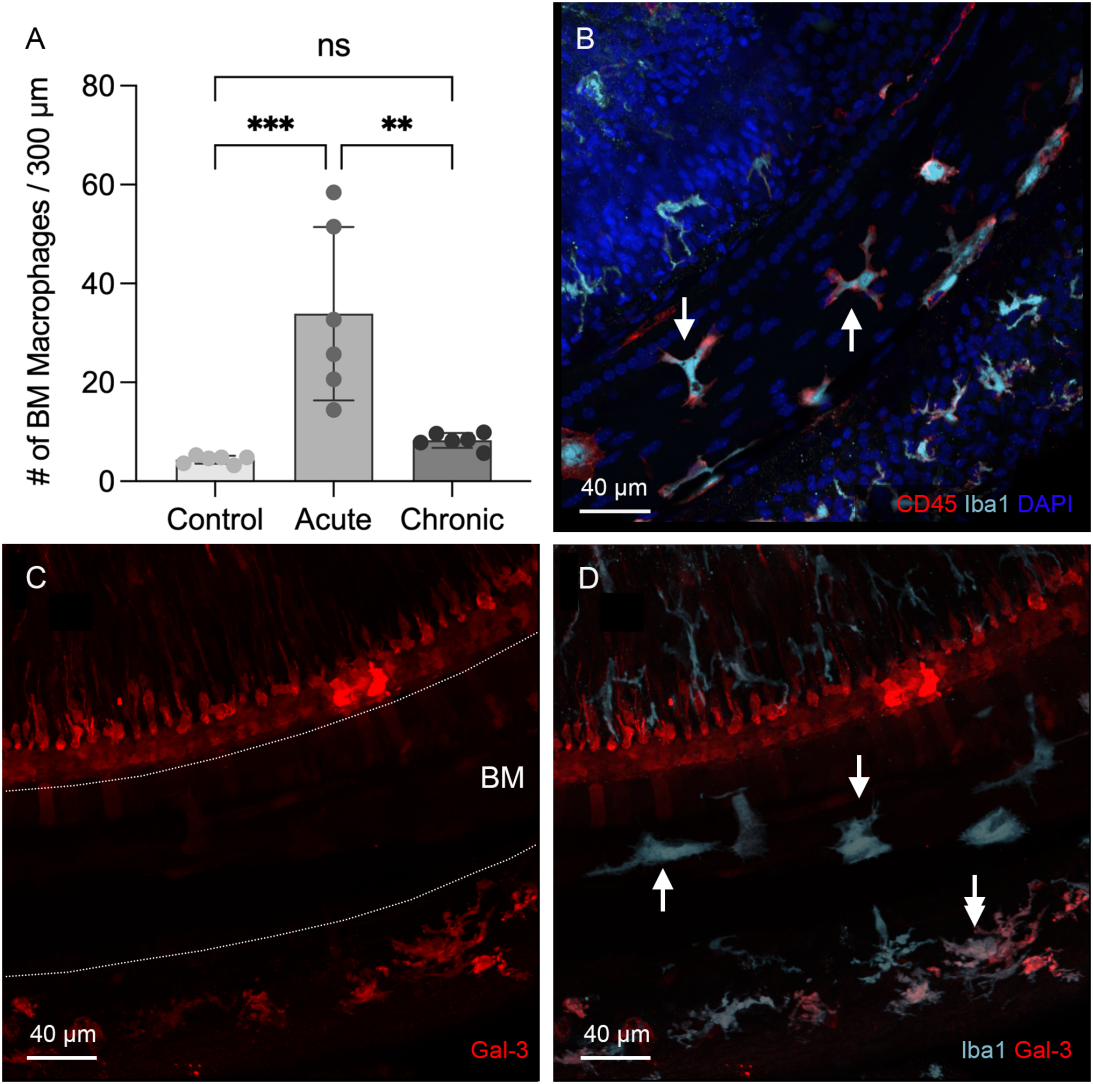
Regression of macrophage activity on the basilar membrane during the chronic phase of cyclodextrin ototoxicity. **(A)** Comparison of the number of BM macrophages among the control, the acute (3–4 days post-treatment), and the chronic phase (6–10 weeks post-treatment) shows that the macrophage number returns to baseline level at the chronic phase (** indicates *p* < 0.01, *** indicates *p* < 0.001). **(B)** Typical morphology of BM macrophages in the middle section of the cochlea at 6 weeks post-treatment. Most BM macrophages exhibit a branched or elongated shape (arrows). The small, round macrophages commonly observed during the acute phase are now rarely present. **(C, D)** Double immunostaining for galectin-3 and Iba1 in the middle section of a cochlea examined six weeks after cyclodextrin treatment. Galectin-3 immunoreactivity was barely detectable in the basilar membrane region (indicated by the area between the two dotted lines in panel **(C)**, despite the presence of Iba1-positive macrophages in this region (arrows in panel **(D)**. Notably, cells medial to the inner hair cells and a subset of macrophages in the lateral wall (indicated by the double arrow) exhibit galectin-3 immunoreactivity. ns, not significant.

Since macrophages were not detected in the organ of Corti during the acute phase of cochlear damage, we extended our observations to the chronic phase of cyclodextrin-induced damage to determine whether macrophages might infiltrate the organ of Corti at a later stage. To this end, we examined cochleae at 6 to 10 weeks after cyclodextrin treatment. For BM macrophages, we observed signs of recovery. Firstly, the number of BM macrophages, which had surged during the acute phase, returned to baseline levels (**Figure 6A**, one-way ANOVA, *F* (2,15) = 14.91, *p* = 0.0003; Tukey’s multiple comparisons test, control *vs*. acute phase, *p* = 0.0004; acute *vs*. chronic phase, *p* = 0.0015, *n*=6 images for each condition). Secondly, the small, round Iba1-positive cells, initially observed in large quantities at 3- and 4-day post-treatment, were now barely detectable (**Figure 6B**). Thirdly, galectin-3 immunoreactivity became almost undetectable in many BM macrophages (**Figures 6C, D**), with only a small subset continuing to exhibit increased expression. Most of these galectin-3 positive macrophages were observed in the lateral wall. These observations suggest that acute cochlear inflammation has largely subsided for BM macrophages during the chronic phase of cyclodextrin ototoxicity.

### Identification of OC macrophages during the chronic phase of cyclodextrin ototoxicity

**Figure 7:**
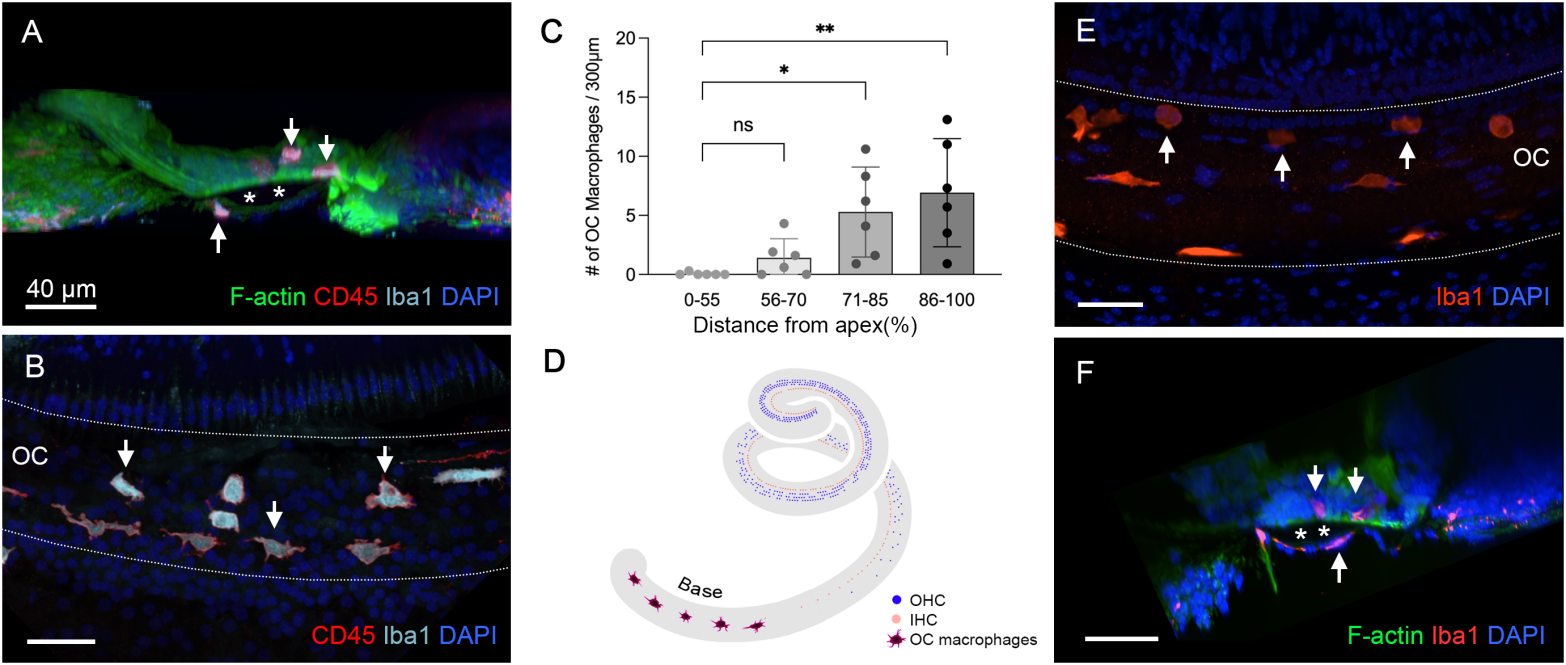
Identification of OC macrophages during the chronic phase of cyclodextrin ototoxicity. **(A)** A side view of the sensory epithelium stained for CD45 and Iba1 from a cochlea examined 6 weeks post-treatment. The two downward arrows at the top indicate OC macrophages, and the upward arrow at the bottom indicates the BM macrophage. The dark space marked by the asterisks is the basement membrane, which separates the OC macrophages from the BM macrophages. **(B)** Top view of the sensory epithelium displaying OC macrophages. This image was created by removing the confocal image layers containing BM macrophages from a 3D stack, thus revealing only OC macrophages. Arrows indicate OC macrophages with different morphologies. **(C)** Quantification of OC macrophages at different regions along the cochlear spirals. (* indicates *p* < 0.05, ** indicates *p* < 0.01) **(D)** A schematic of the cochlear spiral illustrates the distribution of OC macrophages. OC macrophages are primarily confined to the basal region (70–90% from the apex). In contrast, hair cell loss extends across the entire basal and middle regions of the cochlea. **(E)** OC macrophages are present in the cochlea 2 weeks post-treatment (arrows). **(F)** A cross-sectional view of the sensory epithelium confirms that these macrophages are located within the organ of Corti (downward arrows). The asterisks indicate the basement membrane that separates the OC macrophages (downward arrows) from the BM macrophage (upward arrow). ns, not significant; OC, organ of Corti.

Next, we examined the organ of Corti for macrophages. Using 3D confocal imaging and digital cross-sectioning, we identified Iba1- and CD45-positive immune cells within the organ of Corti (**Figure 7A**). This cross-sectional view allows a clear distinction between OC macrophages, located above the basement membrane, and BM macrophages positioned below. Further observations of the organ of Corti along the cochlear spiral showed that some of these cells displayed typical macrophage morphology, characterized by large, irregular shapes, while others were rounded (**Figure 7B**). Their number was typically increased toward the basal end of the cochlea (**Figure 7C**, one-way ANOVA, *F* (3,20) = 6.346, *p* = 0.0029; Dunnett’s multiple comparisons test, 0–55 *vs*. 71-85, *p* =0.0215; 0–55 *vs*. 86-100, *p* = 0.0027, *n*=6 cochlea for each section).

As shown in **Figure 2**, sensory cell loss extended throughout the cochlear spiral in the chronic phase of cyclodextrin ototoxicity, with complete loss in the basal region and nearly complete loss in the middle area of the cochlea. Despite this widespread sensory cell loss, OC macrophages were primarily localized in the basal region (70–90% from the apex, **Figure 7D**). This distribution pattern suggests that their recruitment is not a direct response to the process of OHC degradation, as the OHCs had already been lost during the acute phase of cyclodextrin-induced damage.

To determine the timing of macrophage infiltration into the organ of Corti, we analyzed cochleae at 2 weeks after cyclodextrin treatment. Among the four cochleae examined, only one showed macrophages within the organ of Corti (**Figures 7E, F**). By comparison, all cochleae examined at 6 to 10 weeks post-treatment exhibited OC macrophages. This observation suggests that OC macrophages begin to infiltrate the organ of Corti sometime between 2 and 6 weeks post-treatment.

### Locations of OC macrophages within the organ of Corti

**Figure 8:**
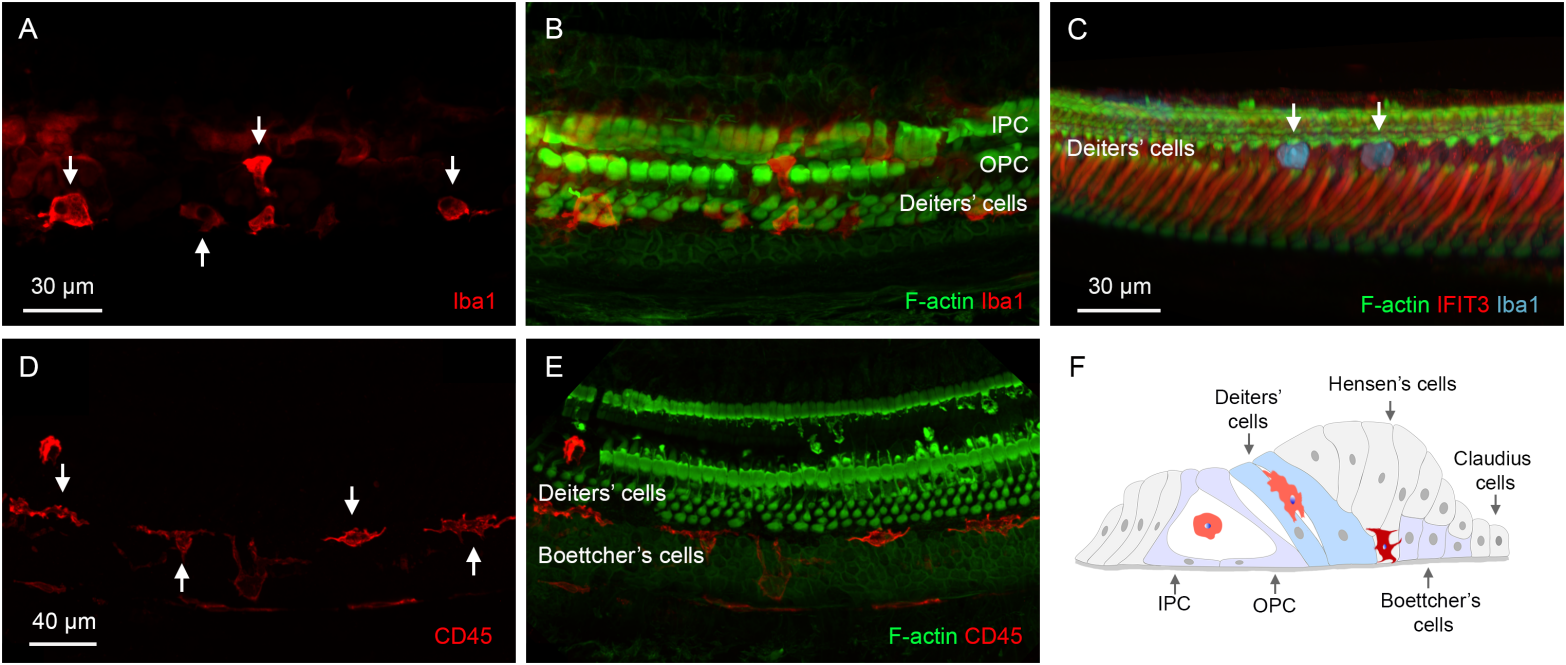
Locations of OC macrophages in the organ of Corti. **(A, B)** A typical location of OC macrophages, illustrated by Iba1 staining, in the region of Deiters’ and pillar cells in a cochlea examined 6 weeks post-treatment. Arrows indicate OC macrophages with irregular shapes. F-actin staining in **(B)** shows the footplates of Deiters’ cells and pillar cells. **(C)** Rounded macrophages (arrows) are present at the top portion of the Deiters’ cell region illustrated by IFIT3 immunostaining, a marker to label the central stalks of Deiters’ cells. Phalloidin staining reveals a loss of F-actin in the cuticular plates of OHCs. **(D, E)** Another common location of OC macrophages is at the junction of Deiters’ and Boettcher’s cells. Macrophages in this region exhibit an elongated morphology, with their long axis aligned with the cochlear spiral. **(F)** A schematic diagram illustrating the two primary locations of OC macrophages. The macrophages highlighted in orange are those located in the region of Deiters’ cells and pillar cells, whereas the macrophage highlighted in dark red is located at the junction between Deiters’ cells and Boettcher’s cells. IPC, inner pillar cell; OPC, outer pillar cell.

OC macrophages were identified at two locations within the organ of Corti. The first location was in the region of Deiters’ and pillar cells (**Figures 8A-C**). OC macrophages in this location typically exhibited irregular shapes, often conforming to the spaces available around them. Some retained a rounded shape, and these cells often appeared within the tunnel of Corti, or in and around areas where supporting cells were absent.

The second location was at the junction between Deiters’ cells and Boettcher’s cells. These OC macrophages typically exhibited an elongated shape and were aligned longitudinally along the cochlear spiral (**Figures 8D, E**). They resided deeply within the organ of Corti, close to the basement membrane. These macrophages were distributed more broadly along the cochlear spiral than those in the region of Deiters’ and pillar cells, but their number was lower. Although the OC macrophages were often found near areas with missing Deiters’ cells, many were also located in regions where Deiters’ cells were present. **Figure 8F** is a schematic showing a cross-sectional view of the organ of Corti, illustrating locations of OC macrophages in the tissue.

### Comparison of immune molecule expression in OC and BM macrophages

**Figure 9:**
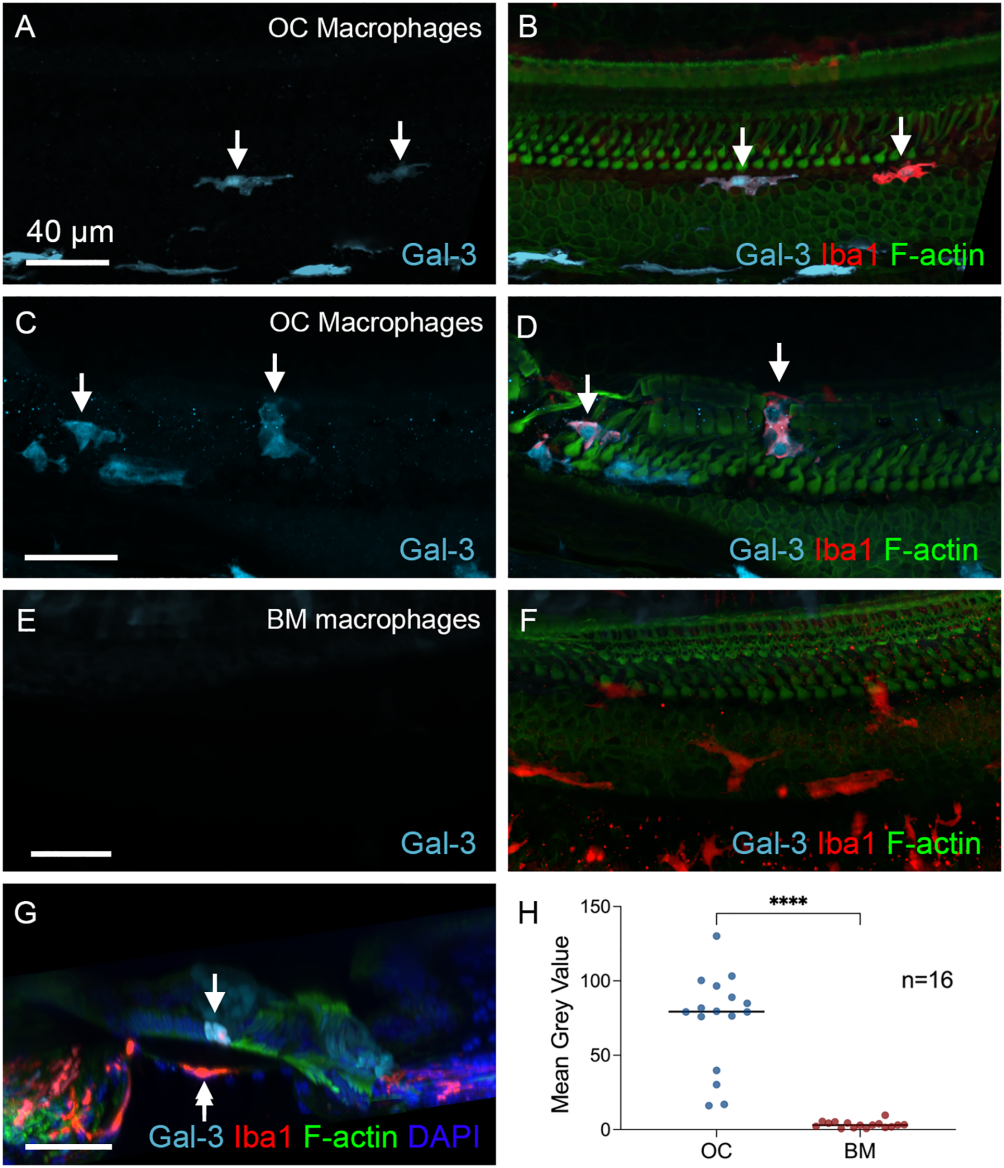
Differences in galectin-3 immunoreactivity between OC and BM Macrophages. **(A, D)** Galectin-3 immunostaining in cochleae examined 6 weeks post-treatment. The images show a basal section of the cochlea (approximately 70-80% from the apex). **(A, B)** show macrophages located at the junctural region between Deiters’ cells and Boettcher’s cells. **(C, D)** show macrophages within the region of Deiters’ cells and pillar cells. Arrows point to the macrophages with strong galectin-3 immunoreactivity. **(E)** and **(F)** Images show BM macrophages of the same regions. These macrophages show no detectable galectin-3 immunoreactivity. **(G)** Cross-sectional view of the sensory epithelium. The arrow indicates an OC macrophage with strong galectin-3 immunoreactivity, whereas the double-arrow identifies a galectin-3-negative BM macrophage. **(H)** Quantitative comparison of the mean gray value of galectin-3 fluorescence demonstrates significantly stronger immunoreactivity in OC macrophages than in BM macrophages. **** indicates *p* < 0.0001. OC, OC macrophages; BM, BM macrophages. *n*=16 macrophages from 6 cochleae.

To assess the functional state of OC macrophages during the chronic stage, we examined galectin-3 expression levels. Our analysis revealed that all macrophages within the organ of Corti exhibited strong galectin-3 immunoreactivity. This included those located between Deiters’ cells and Boettcher’s cells (**Figures 9A, B**) as well as those positioned in the region of Deiters’ and pillar cells (**Figures 9C, D**). In contrast, BM macrophages showed a lack of galectin-3 immunoreactivity (**Figures 9E, F**). A cross-sectional view of the organ of Corti clearly demonstrated the presence of galectin-3 positive macrophages within the organ of Corti, while BM macrophages were galectin-3 negative (**Figure 9G**). Quantitative assessment of galectin-3 immunoreactivity further supported this observation (**Figure 9H**, two-tailed unpaired Student’s *t* test, *t* (30) = 8.770, *p* < 0.0001, *n*=16 macrophages from 6 cochleae).

**Figure 10:**
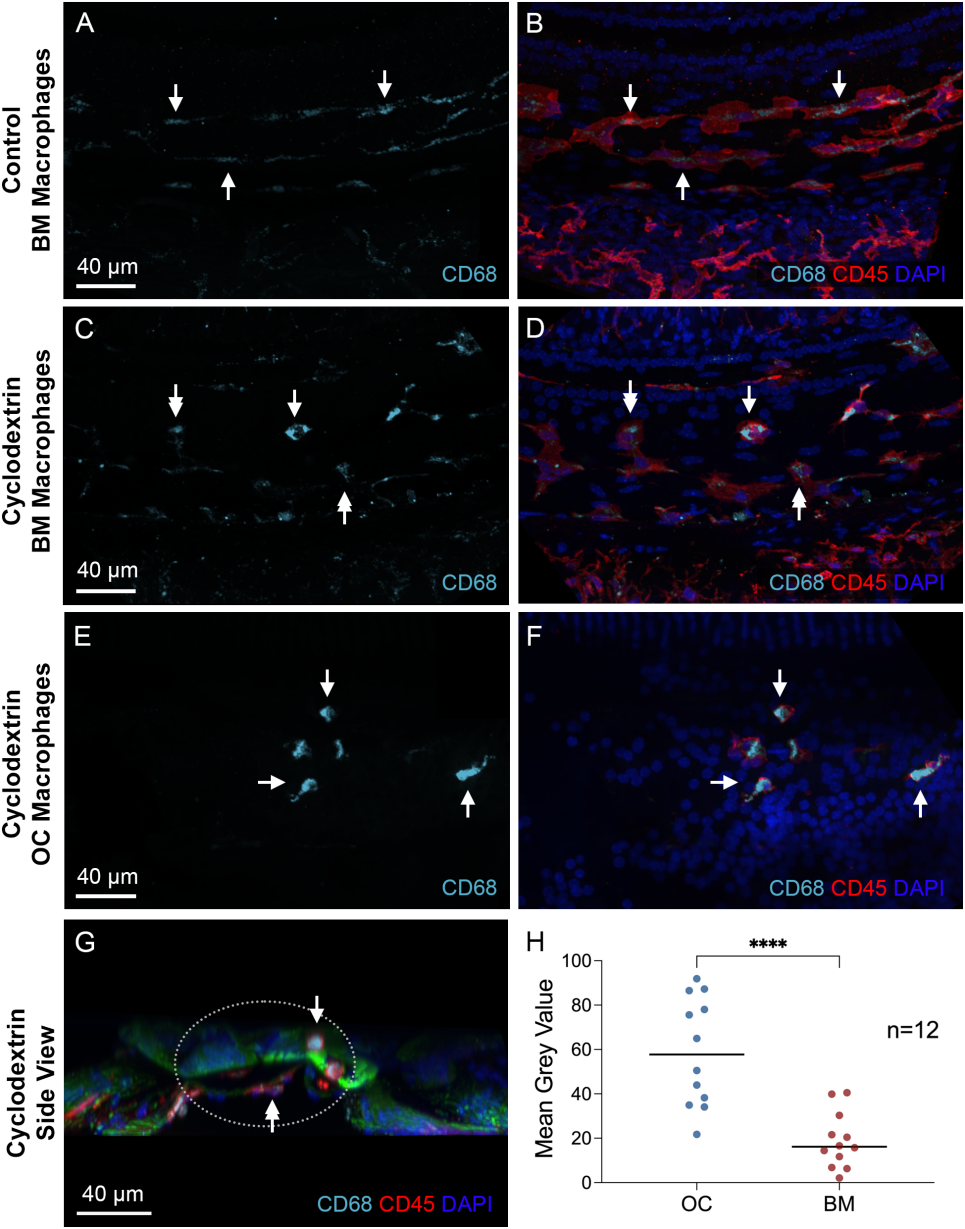
Differences in CD68 immunoreactivity between OC and BM macrophages. **(A, B)** Typical images of CD68 immunoreactivity in BM macrophages located in the middle region of a normal control cochlea. Arrows indicate BM macrophages with weak CD68 immunoreactivity. **(C, D)** CD68 immunoreactivity in BM macrophages 6 weeks after cyclodextrin treatment. These macrophages exhibit varying levels of CD68 immunoreactivity in their cytoplasm. Some display levels comparable to those observed in control cochleae (double arrows), while others show stronger immunoreactivity (single arrow). **(E, F)** CD68 immunoreactivity in OC macrophages of a cyclodextrin-treated cochlea examined 6 weeks after treatment. Although variable among individual cells, all OC macrophages display stronger CD68 immunoreactivity (arrows), suggesting enhanced CD68 expression. **(G)** A cross-sectional view of the sensory epithelium from a cyclodextrin-treated cochlea shows the difference in CD68 immunoreactivity between OC and BM macrophages. The dotted ellipse highlights the region containing both OC and BM macrophages. Notably, CD68 immunoreactivity is stronger in OC macrophages (single arrow) compared to BM macrophages (double arrow). **(H)** Quantitative analysis of the mean gray level of CD68 immunoreactivity in cyclodextrin-treated cochleae. OC macrophages exhibit significantly higher CD68 immunoreactivity compared to BM macrophages. **** indicates *p* < 0.0001. OC, OC macrophages; BM, BM macrophages. *n*=12 macrophages from 3 cochleae.

Next, we examined the immunoreactivity of CD68, a glycoprotein associated with macrophage phagocytic activity (44–47). Under normal conditions, BM macrophages showed weak CD68 immunoreactivity (**Figures 10A, B**). In contrast, cyclodextrin-treated cochleae exhibited variable levels of CD68 expression among BM macrophages, with some displaying increased CD68 immunoreactivity after treatment, while others displayed levels comparable to those observed under normal conditions (**Figures 10C, D**). However, for OC macrophages, CD68 immunoreactivity was significantly elevated (**Figures 10E, F**). A side view of the organ of Corti reveals strong CD68 immunoreactivity in OC macrophages (**Figure 10G**). To validate these findings, we measured the gray level of CD68 immunoreactivity and found statistically significant differences in the gray levels between OC macrophages and BM macrophages (**Figure 10H**, two-tailed unpaired Student’s *t* test, *t* (22) = 5.044, *p* < 0.0001, *n*=12 macrophages from 3 cochleae). Together, these observations suggest that OC macrophages exhibit a different functional state compared to BM macrophages.

### Chronic pathogenesis of supporting cells

**Figure 11:**
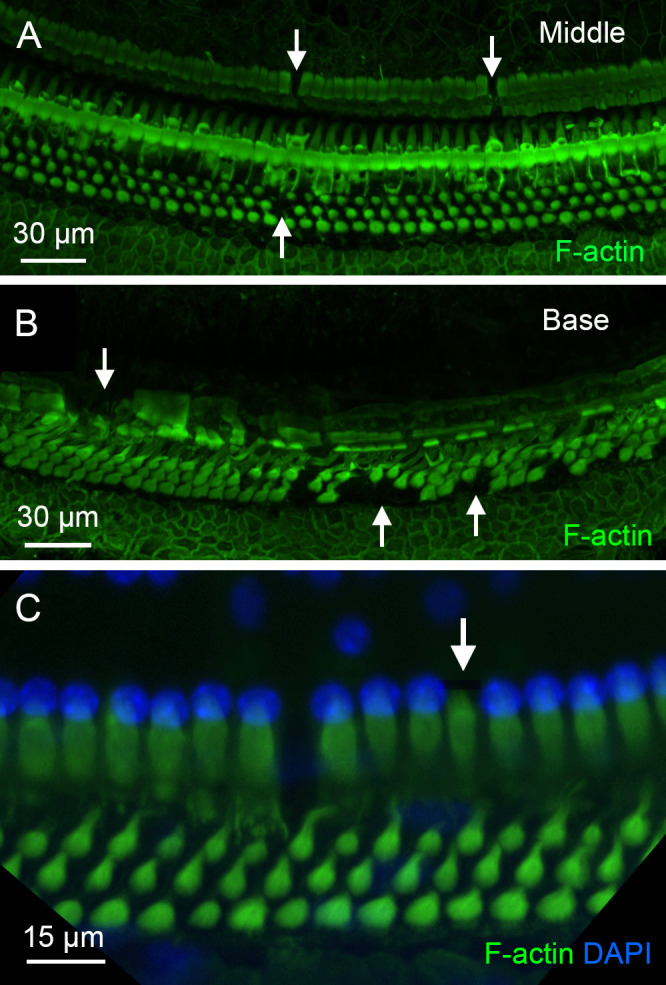
Pathological changes in supporting cells during the chronic phase of cyclodextrin ototoxicity. **(A, B)** F-actin staining of the organ of Corti reveals sporadic loss of supporting cells in the middle region **(A)** and a more pronounced loss in the basal region **(B)** of a cochlea examined at 6 weeks after cyclodextrin treatment. Arrows indicate areas where supporting cells are absent. **(C)** Evidence of active degradation in supporting cells. The arrow points to an inner pillar cell exhibiting retained F-actin staining in the footplate but lacking a nucleus, suggestive of ongoing degeneration.

The finding of activated OC macrophages prompted us to examine the pathological state of the organ of Corti to better understand the conditions that trigger macrophage activity in this region. As shown in **Figure 2**, OHC loss had occurred during the acute phase of cyclodextrin ototoxicity (1–4 days after cyclodextrin treatment), with no significant progression observed in the chronic stage. To further assess the structural integrity of the organ of Corti, we focused on supporting cells, including Deiters’ cells, outer pillar cells, and inner pillar cells, because of their close association with OHCs. Phalloidin staining was used to illustrate the footplates of Deiters’ cells and pillar cells, allowing for the identification of missing cells. Sporadic loss of supporting cells was observed in the apical and middle regions of the cochlea (approximately 0–70% distance from the apex, **Figure 11A**), while significant loss occurred in the basal region (approximately 70–100% distance from the apex, **Figure 11B**). Notably, some supporting cells with relatively intact staining in their footplate were observed to have missing nuclei (**Figure 11C**), indicating that these cells were undergoing active degradation.

**Figure 12:**
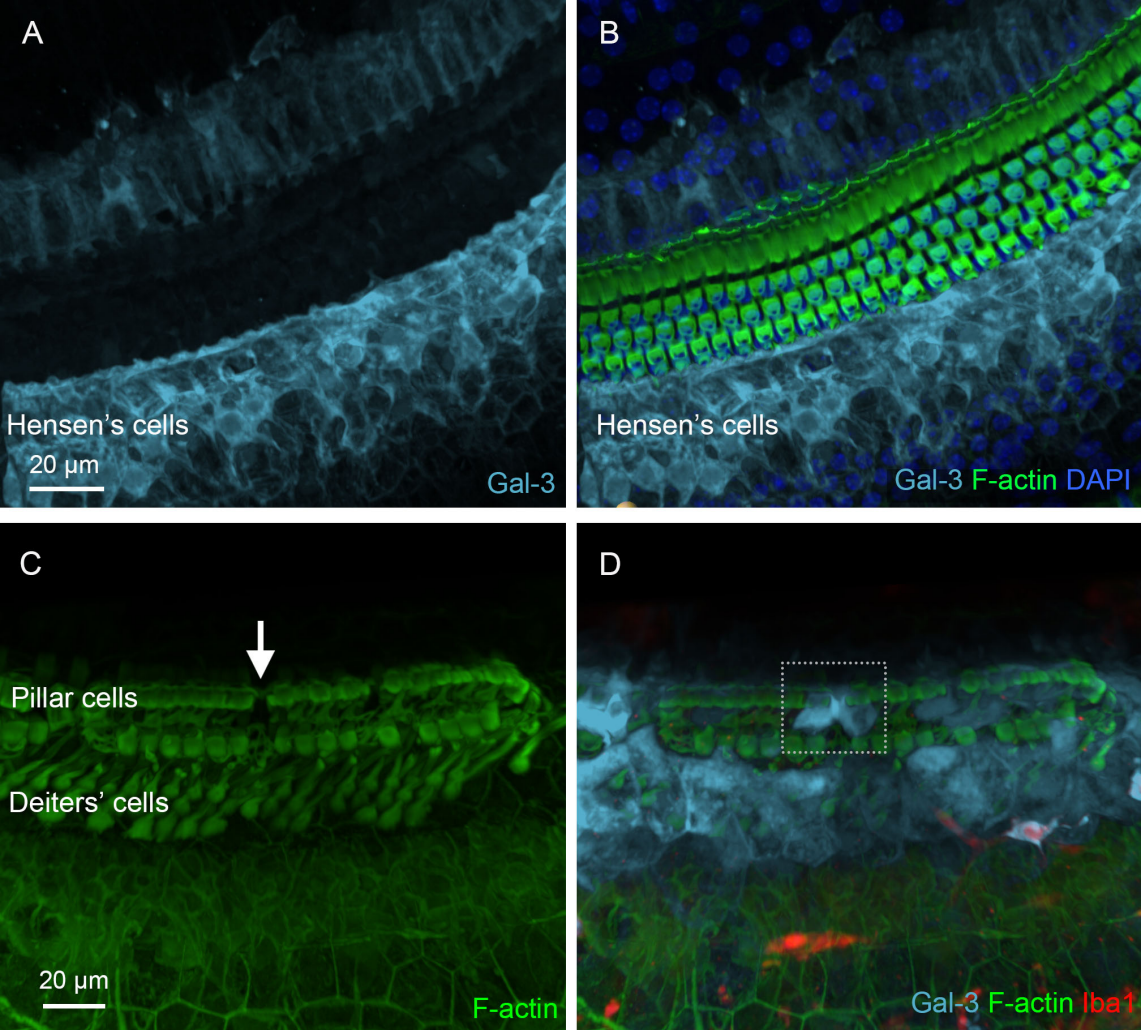
Galectin-3 expression in supporting cells under normal conditions and during the chronic phase of cyclodextrin ototoxicity. **(A)** Galectin-3 immunoreactivity in supporting cells under normal conditions. **(B)** The overlay of panel *A* with F-actin staining to illustrate the organ of Corti. Hensen’s cells exhibit strong galectin-3 immunoreactivity, whereas pillar cells and Deiters’ cells show little, if any, galectin-3 immunoreactivity. **(C, D)** Elevated galectin-3 immunoreactivity in supporting cells adjacent to an area of missing supporting cells. The arrow in **(C)** indicates a region with a missing pillar cell, as evidenced by the absence of F-actin staining. The dotted boxes in **(D)** highlight increased galectin-3 immunoreactivity in supporting cells surrounding the region of the missing pillar cell. As expected, these cells lack Iba1 immunoreactivity.

In addition to analyzing missing supporting cells, we examined galectin-3 expression in surviving supporting cells, as this molecule not only serves as a marker of macrophage activation but also plays a role in stress responses and tissue remodeling in non-immune cells (36–38, 43, 48). In the normal organ of Corti, galectin-3 immunoreactivity was localized to Hensen’s cells and in the region medial to IHCs (**Figures 12A, B**). In contrast, sensory cells, Deiters’ cells, and pillar cells lacked detectable galectin-3 immunoreactivity. However, in the chronic phase of cyclodextrin-induced damage, strong galectin-3 immunoreactivity was observed in numerous supporting cells adjacent to the areas where supporting cells were missing. As shown in **Figures 12C, D**, the region of a missing pillar cell is surrounded by elevated galectin-3 immunoreactivity in the adjacent pillar cell. Notably, this change was observed in many, but not all, regions near missing cells, suggesting that enhanced expression may occur only at a specific stage of supporting cell pathogenesis or during the repair process. Collectively, our observations revealed prolonged pathogenesis of supporting cells during the chronic phase of cyclodextrin ototoxicity.

### Macrophages in the region of Claudius cells

**Figure 13:**
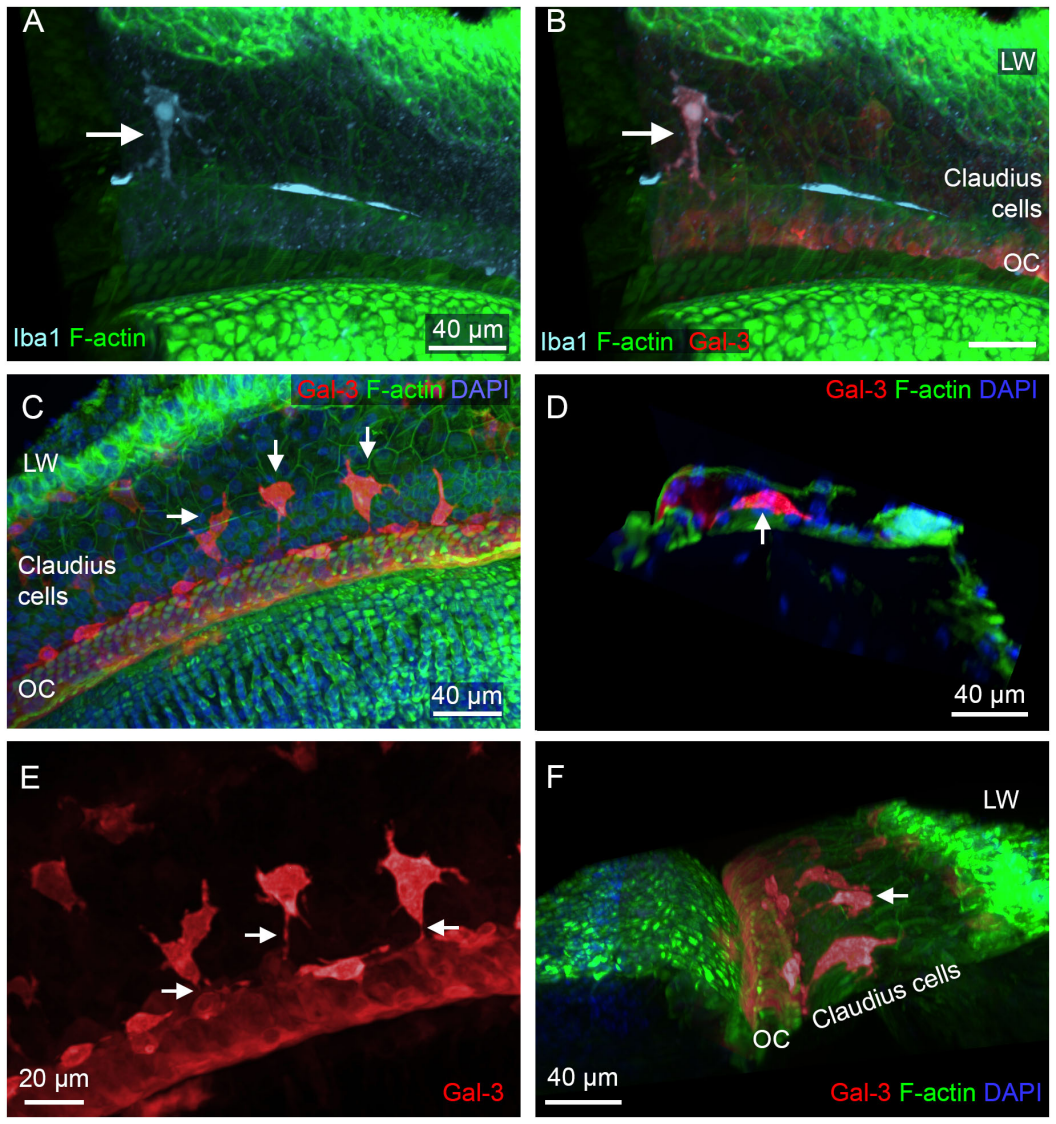
Macrophages in the Claudius cell region in cyclodextrin-treated cochleae. **(A, B)** Images show a macrophage in the Claudius cell region in a cochlea examined 6 weeks after cyclodextrin treatment. This cell displays Iba1 **(A)** and galectin-3 immunoreactivities **(B)**. **(C)** Macrophages with strong galectin-3 immunoreactivity display irregular shapes (arrows). **(D)** A side view of the sensory epithelium shows a macrophage (arrow) located beneath Claudius cells. **(E)** Enlarged view of macrophages in the Claudius cell region. Macrophages display projections toward the organ of Corti (arrows). **(F)** A 3D view of the organ of Corti showing macrophages in the Claudius cell region. The macrophages exhibit radial processes (arrow) directed toward the organ of Corti. LW, lateral wall; OC, organ of Corti.

In addition to the locations described above for OC macrophages, we identified Iba1-positive macrophages in the region lateral to the organ of Corti (**Figures 13A, B**). These cells displayed galectin-3 immunoreactivity. We examined their morphological characteristics using galectin-3 staining, which provided clearer visualization of their morphology (**Figures 13C-F**). We found that these cells were located beneath Claudius cells and had an irregular shape. Their numbers varied across the cochleae examined, ranging from none to numerous. Interestingly, these cells typically extended their processes toward the organ of Corti, as shown in an enlarged view of macrophages (**Figure 13E**), and a 3D side view of the organ of Corti (**Figure 13F**). These observations suggested that the cells were migrating from the lateral wall toward the organ of Corti.

### OC macrophages in aging cochleae

**Figure 14:**
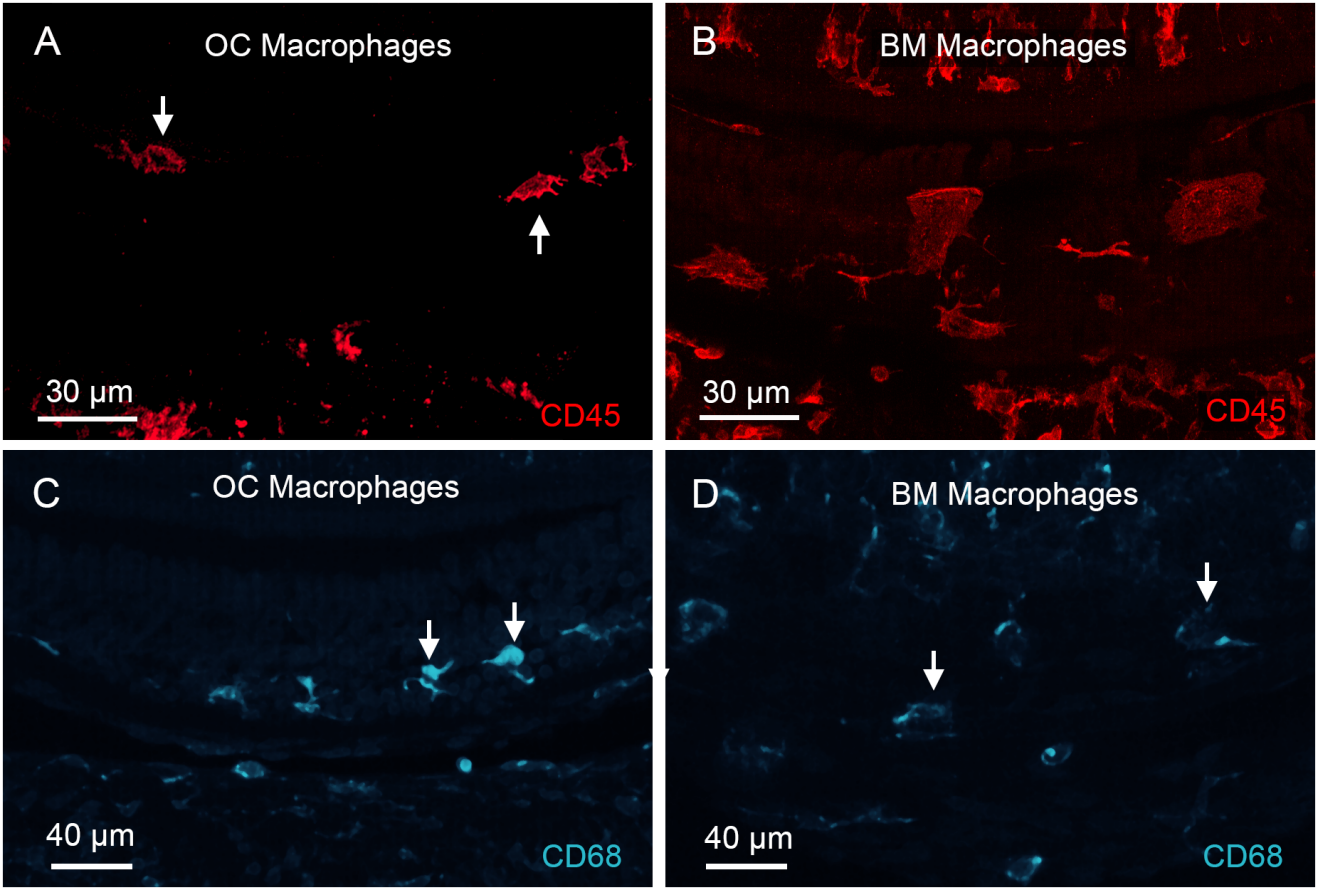
Identification of OC macrophages in aging cochleae. **(A, B)** CD45 immunolabeling of the basal region of a cochlea collected from a mouse at 19 months of age. **(A)** shows a confocal image containing only the organ of Corti layer, excluding the basilar membrane, to illustrate OC macrophages. The arrows point to OC macrophages. Panel **(B)** shows a confocal image of the basilar membrane layer, excluding the organ of Corti, to illustrate BM macrophages. Notice that OC macrophages appear smaller with irregular or elongated shapes and multiple processes, whereas BM macrophages exhibit a larger, flat morphology. **(C, D)** CD68 immunoreactivity in OC and BM macrophages in the aging cochlea examined at 19 months of age. OC macrophages display stronger CD68 immunoreactivity than BM macrophages. Arrows in images point to macrophages.

Given our finding of OC macrophages in the chronic phase of cyclodextrin-induced ototoxicity, we sought to determine whether this phenomenon also occurs in other chronic pathological conditions. In a previous study on C57BL/6J mice, a strain that is predisposed to early-onset age-related sensory cell loss due to the *Cdh23* mutation (49, 50), we observed the presence of BM macrophages, but did not detect any OC macrophages, in mice up to 12 months of age (31). In the current study, we extended the investigation to mice aged 17 to 19 months and observed the presence of macrophages in the organ of Corti. Like the OC macrophages seen in cyclodextrin-treated cochleae, these cells were also located in the basal region of the cochlea, where complete sensory cell loss had occurred. These macrophages exhibited irregular shapes with multiple processes (**Figure 14A**), differing from the large, flat BM macrophages observed in the same region along the cochlear spiral (**Figure 14B**). To investigate whether OC macrophages exhibit distinct functional states compared to those outside the organ of Corti, we observed CD68 immunoreactivity in both OC macrophages and BM macrophages, with differing expression levels (**Figures 14C, D**); OC macrophages exhibited stronger immunoreactivity. These findings align with those observed in cyclodextrin-treated cochleae.

## Discussion

The current study aims to characterize OC macrophages and identify pathological conditions that trigger their recruitment to the organ of Corti. Consistent with previous reports, we confirm that macrophages are absent from the organ of Corti in mouse cochleae under normal conditions and during the acute phase of cochlear damage (8, 10, 40). Importantly, we found that macrophages emerge within the organ of Corti during the chronic phase of cochlear pathogenesis. These cells exhibit an activated state and display a distinct functional profile compared to macrophages outside the organ of Corti. This suggests that OC macrophages appear to be associated with supporting cell pathogenesis rather than sensory cell pathogenesis. The presence of OC macrophages was observed in both ototoxicity and age-related degeneration models examined in this study. Although their precise roles remain unclear, our results suggest that OC macrophages may influence the fate of supporting cells by contributing to their survival, degeneration, or both, during chronic cochlear pathogenesis.

### Macrophages in the acute stage of cochlear damage

Sensory cells in the cochlea are highly vulnerable to pathological insults, and the removal of fatally damaged sensory cells is crucial for tissue repair. Previous studies have shown that cochlear sensory cell damage triggers inflammation, leading to the infiltration of circulating monocytes into the cochlea (1, 8, 51). These observations suggest that cochlear macrophages may play a direct role in clearing damaged sensory cells. However, although we also observed a marked increase in cochlear macrophages during the acute phase of damage, all macrophages at this stage were located outside the organ of Corti. Absence of macrophages within the organ of Corti when a large number of OHCs are dying raises a critical question of whether cochlear macrophages directly contribute to the clearance of damaged OHCs during the acute phase of cochlear pathogenesis. One explanation is that macrophages neighboring the organ of Corti might extend their processes through the basement membrane to reach damaged OHCs (52, 53). However, careful examination of our confocal images failed to reveal any trans-basilar membrane processes. Based on these findings, we suspect that cochlear macrophages are unlikely to be directly involved in clearing damaged sensory cells within the organ of Corti during the acute stage of injury.

If macrophages do not directly engulf dying sensory cells, how is their debris cleared from the organ of Corti? Previous studies have shown that supporting cells are capable of phagocytosing dead hair cells (21, 22, 52). Our prior research demonstrated that supporting cells express toll-like receptors (54), which are involved in detecting molecules released from damaged tissue and activating immune responses. In addition to Toll-like receptors, retinoic acid-inducible gene-I-like receptors, another class of cytoplasmic pattern recognition receptors, have been identified in supporting cells, including Hensen’s cells and Claudius cells (15). These receptors play an important role in cochlear immune responses to viral infections. Therefore, it is likely that during the acute stage of cochlear damage, supporting cells play a role in clearing damaged sensory cells. While macrophages are not present within the organ of Corti, they may still play supportive roles. For example, cell debris can be released from the organ of Corti and subsequently cleared by macrophages residing outside the organ of Corti. Our previous scanning electron microscopy studies have shown such macrophage-mediated debris clearance in the scala tympani (53). Thus, macrophages, though not directly present in the organ of Corti, may still contribute to repair processes in the organ of Corti. Clarification of this question requires further investigation.

### Pathological conditions for the recruitment of OC macrophages into the cochlea

A key finding of the current study is the identification of pathological conditions that promote the recruitment of macrophages into the organ of Corti. In cyclodextrin-treated mice, we observed macrophage accumulation within the organ of Corti during the chronic stage of cochlear damage. Notably, these OC macrophages were predominantly located at the basal end of the cochlea, where all sensory cells had already been lost. To determine whether findings from the acute ototoxicity model could be generalized to other pathological conditions, we examined macrophage activity in the aging cochlea. Our observations revealed that macrophage recruitment into the organ of Corti is not limited to the chronic phase following acute damage but can also occur in the setting of chronic cochlear degeneration alone. Notably, OC macrophages were observed exclusively in regions of the organ of Corti where all OHCs had been lost.

The finding that OC macrophages were found exclusively in regions lacking surviving OHCs differs from previous observations in human cochleae, where OC macrophages were observed near hair cells with preserved morphology (5, 30). Several factors could contribute to this discrepancy. First, the two studies used different species. The current study used mice, whereas the previous one examined human tissues. Second, the etiology of cochlear damage differed: our study used cyclodextrin treatment, while the previous study examined cochleae from patients with posterior cranial fossa meningiomas. These differences in species and damage mechanisms may affect macrophage activity in the organ of Corti, potentially explaining the different findings.

The present study sought to determine the pathological conditions under which macrophages are recruited into the organ of Corti following sensory cell loss. Several pieces of evidence suggest that the presence of OC macrophages is associated with supporting cells. First, we found that supporting cells at the basal end of the cochlea exhibited signs of pathogenesis while simultaneously engaging in tissue repair at the chronic phase of cochlear damage. Specifically, some supporting cells lacked nuclei, indicating active degradation. Additionally, we observed that supporting cells located near areas of missing supporting cells showed increased galectin-3 expression, suggesting an active response to supporting cell damage. These observations suggest that supporting cell pathogenesis persists at this chronic stage. Second, we found that OC macrophages were located in regions where supporting cells were either missing or activated, suggesting that these macrophages responded to supporting cell pathogenesis. While the precise roles of OC macrophages in supporting cell damage and repair remain unclear, the strong expression of galectin-3 suggests that these macrophages were in an activated state. Moreover, elevated CD68 expression in OC macrophages, a phagocytosis marker, indicates these macrophages may be involved in clearing damaged supporting cells. Future studies are needed to determine whether macrophages contribute to or protect against supporting cell loss.

### Source of OC macrophages

An important question arising from this study is the origin of OC macrophages. Since these macrophages are not present within the organ of Corti under normal conditions, they must migrate from surrounding tissues in response to pathological conditions. We suspect that lateral wall macrophages contribute to the OC macrophage population, as the lateral wall is a major site of macrophage accumulation during cochlear inflammation (55–57). To investigate this, we carefully examined the area surrounding the organ of Corti. We identified macrophages positioned beneath Claudius cells, in the region between the lateral wall and the organ of Corti. Like other OC macrophages, these macrophages were confined to the basal end of the cochlea. Interestingly, they often oriented their long axis toward the organ of Corti, suggesting directed migration. Given their location between the lateral wall and the organ of Corti, we suspect these macrophages may serve as a source of OC macrophages by migrating through the subcellular space beneath the Claudius cells to reach the organ of Corti. Future studies employing dynamic observation techniques will be essential to confirm this hypothesis.

### In summary

This study investigated macrophage responses to cochlear damage induced by cyclodextrin. We found that acute cyclodextrin ototoxicity led to rapid sensory cell loss. While macrophages were recruited to the cochlea during the acute stage of ototoxic damage, they remained outside of the organ of Corti. In contrast, macrophages infiltrated the organ of Corti during the chronic stage of cyclodextrin pathogenesis, where they exhibited an activated state marked by strong galectin-3 and CD68 expression. Notably, the presence of OC macrophages was spatially correlated with supporting cell pathogenesis. Similar patterns of OC macrophage activity were also observed in aging cochleae. Overall, our study suggests the involvement of OC macrophages in supporting cell pathogenesis and repair. These findings provide valuable insights for future strategies focused on modulating macrophage activity to mitigate tissue damage and promote repair in the cochlea after injury.

## Data Availability

The original contributions presented in the study are included in the article/supplementary material. Further inquiries can be directed to the corresponding author.
